# Assessing teacher education and professional development needs for the implementation of integrated approaches to STEM education

**DOI:** 10.1186/s40594-017-0068-1

**Published:** 2017-06-17

**Authors:** David J. Shernoff, Suparna Sinha, Denise M. Bressler, Lynda Ginsburg

**Affiliations:** 1 Center for Mathematics, Science, and Computer Education, Rutgers University, New Brunswick, NJ USA; 2 Department of School Psychology, Graduate School of Applied and Professional Psychology, Rutgers University, New Brunswick, NJ USA

## Abstract

**Background:**

Given the growing interest in, and relevance of, integrated approaches to STEM (science, technology, engineering, and mathematics) education, there is an urgent desire to understand the challenges and obstacles to developing and implementing integrated STEM curricula and instruction. In this article, we present phase 1 of a two-phase needs assessment study to identify challenges and needs of promoting integrated approaches in STEM education. Utilizing a key informant approach, 22 K-12 teachers and four administrators selected as potential leaders in STEM education in an unidentified state on the East Coast of the USA were interviewed. Participants were asked to identify challenges and perceived supports to conduct integrated STEM education. Questions were open-ended in order to inform a larger, state-wide questionnaire study in phase 2 to be reported subsequently and were qualitatively coded.

**Results:**

Several distinctive themes were identified as described by teacher participants when discussing challenges and obstacles of implementing integrated STEM education, as well as supports that would be most helpful in overcoming them. Participants also provided specific suggestions for teacher education needed to support integrated STEM education.

**Conclusions:**

Preliminary findings suggest that many teachers are interested in integrated approaches to STEM, but do not believe they are well prepared to implement them. Teachers and administrators also suggest that adequate preparation in integrated STEM would entail a considerable rethinking and redesigning of pre-service courses and in-service workshops. Findings provide a starting point for better understanding teacher needs in integrated STEM and a springboard for further study.

## Background

Integrated approaches to STEM (science, technology, engineering, and mathematics) education are increasingly popular, but remain challenging and elusive. There is much hope that integrated approaches to STEM education can help the next generation of students to solve real-world problems by applying concepts that cut across disciplines as well as capacities of critical thinking, collaboration, and creativity (Burrows and Slater 2015). However, most teachers have received training in only one discipline (Honey et al. 2014), and most schools and classes at all levels still have separate departments and class periods for the STEM subjects. Therein lies a significant challenge for educators and administrators interested in promoting integrated STEM.

In this article, we present phase 1 of a two-phase integrated STEM education and teacher professional development (PD) needs assessment study to identify challenges and needs of promoting integrated STEM education. The study focuses on challenges of fostering integrated STEM education and potential supports needed to overcome them in an unidentified state on the East Coast of the USA. We sought to learn what supports would be most helpful in the effort to integrate STEM disciplines in curricula and instruction, especially in terms of pre-service education and continuing professional development (e.g., teacher “in-service” workshops). Needs assessments are important for data-driven decision making to benefit a maximum number of districts, schools, teachers, and students with available resources (Nagle and Gagnon 2008). A primary motivator of the present study was to inform the resource allocation of STEM education centers and the state Department of Education (DoE) in the state examined. The study occurred in the context of a partnership between one such center and the state DoE.

In phase 1 of the study, we interviewed a selection of K-12 STEM teachers and math/science supervisors throughout the state who participated in a state-sponsored initiative in integrated STEM. These *key informant* interviews are intended to inform a larger, statewide questionnaire in phase 2 of the study to be reported subsequently upon completion.

### Why integrated STEM now?

Education in science, mathematics, engineering, and technology (STEM) is widely recognized as a pressing state and national priority (Honey et al. 2014). Excellence in STEM education can impact jobs, productivity, and competitiveness in multiple sectors and fields including health, technological innovation, manufacturing, the distribution of information, political processes, and cultural change (Asunda 2014; Peters 2006). Innovation in STEM fields drives not only economic growth, but also the quality of life. STEM-related jobs are expected to grow at 17% compared to 9.8% for non-STEM jobs (Langdon et al. 2011). At the same time, the pipeline into STEM jobs can be described as leaky: in the USA, only 75% of students who focus or excel in STEM subjects in K-12 enter STEM majors; 38% of STEM majors do not graduate with a STEM degree; 43% of STEM graduates do not go on to work in a STEM occupation; and 46% of STEM workers with a bachelors in STEM leave STEM fields for higher paying managerial roles (Georgetown University Center on Education and the Workforce, n. d.). While there are different perspectives on the extent to which shortages in qualified STEM workers are real or perceived (Charette 2013; The Royal Society Science Policy Centre 2014), there is increasing and nearly universal concern over the quality of education and skill development in STEM subjects.

While these factors have led to dramatic gains in focus on STEM education, the vision of STEM education has also evolved. A new vision calls for STEM to be learned and taught in integrated ways with interdisciplinary methods (US Department of Education and Office of Innovation and Improvement 2016). Because solutions to most global challenges concerning energy, health, and the environment (e.g., climate change, sustainability) require an interdisciplinary (and frequently, international) perspective involving math, science, and technology, recent reforms such as the Next Generation Science Standards (NGSS) and Common Core State Standards for Mathematics (CCSSM) advocate for intentionally integrating instruction and curricula by providing stronger connections among the STEM disciplines. The NGSS is being adopted by many states in the USA including the state in which our study is conducted, and calls for students to learn concepts cutting across the STEM disciplines, science and engineering practices, as well as disciplinary core ideas. It also elevates the need for teaching engineering in K-12, which can drive the solving of problems requiring science and mathematics. Supported by empirical evidence that the engineering design process can be an effective way to facilitate and sustain the integration of concepts from multiple STEM disciplines (e.g., Estapa and Tank 2017; Guzey et al. 2016), the NGSS recognizes the engineering design process as both an important practice and disciplinary core idea that students should master (Moore et al. 2014).

Thus, integrated STEM education has quickly become a meta-discipline, one with a focus on innovation, designing solutions, and leveraging technology (Kelley and Knowles 2016). Students are expected to engage in a rigorous curriculum, with instruction and assessment in math and science inquiry, as well as engineering design (Kelley and Knowles 2016). There is hope that such approaches, which are frequently project-based and engaging, will motivate more students into pursuing STEM careers (Stohlmann et al. 2012). For many, “STEM education” has now come to mean “integrated STEM” education.

### Defining integrated STEM education

In this study, we seek to solicit the current understandings and definitions of integrated STEM in the educational community in order to better understand the need for definitional clarity. A variety of definitions of integrated STEM education have been proposed, but there remains little clear consensus. Most definitions revolved around the integration of one or more STEM disciplines in the teaching and learning process. For example, Sanders (2009) defined integrated STEM education as “approaches that explore teaching and learning between/among any two or more of the STEM subject areas, and/or between a STEM subject and one or more other school subjects” (p. 21). Moore et al. (2014) add that the combining of disciplines is “based on connections between the subjects and real world problems," and more specifically entails, “an effort by educators to participate in engineering design as a means to develop technologies that require meaningful learning and an application of mathematics and/or science” (p. 38). In addition to the teaching of two or more STEM domains, Kelley and Knowles (2016) further characterize integrated STEM as “bound by STEM practices within an authentic context for the purpose of connecting these subjects to enhance student learning” (p. 3). Stohlmann et al. (2012) suggest that integrated STEM education is an effort to combine the STEM disciplines into one class, but clarify that it can involve multiple classes and need not involve all four STEM disciplines. This is not a trivial clarification, because one common question is whether all four STEM disciplines need be integrated for instruction to be considered integrated STEM.

Other definitions include a more expanded “vision” of what good integrated STEM education would “look like,” far transcending the integration of disciplines. For example, the Dayton Regional STEM Center (2011) has promoted a principle-based framework for defining quality STEM and integrated STEM education based on 10 components. In addition to some principles of STEM discipline integration, they include principles, such as “the potential for engaging students from diverse backgrounds,” “quality of the cognitive tasks,” and “connections to STEM careers.”

Many schools focusing on integrated STEM also integrate non-STEM disciplines such as social studies/science into STEM disciplines and vice versa. When integration includes the arts, this has been referred to as STEAM or integrated STEAM. This study was confined to integrated STEM education for the primary reason that it was situated in a STEM academy hosted by the state’s Department of Education (DoE) in the summer of 2016, which did not incorporate the integration of non-STEM subjects. The state DoE’s current conceptualization of STEM education is characterized by integrated approaches. At the STEM Academy, the DoE crafted and offered the following definition to its participants: “STEM education is the use of science, technology, engineering, mathematics, and their associated practices, to create student-centered learning environments in which students investigate and engineer solutions to problems, and construct evidence-based explanations of real world phenomena. Evidence-based STEM education promotes creativity and innovation while developing critical thinking, collaboration, and communication skills while students seek explanations about the natural world to improve the built world”. This was a common conceptualization of integrated approaches to STEM education that was offered to all participants of the present study. However, it should be noted that the same state has assembled a growing task force, led by its School Boards Association, in iSTEAM (i.e., “integrated STEAM”). Thus, the desire to include other subjects in integrated STEM approaches is very much the current trend within the state, but it was not addressed in the current study.

### A conceptual framework for integrated STEM

For supports of integrated STEM education to gain sufficient momentum, a conceptual framework that goes beyond a simple definition is needed and should include the rationale, goals, intended outcomes, components, and how the components interact. A conceptual framework can also help to build a research agenda to inform stakeholders and to realize the full potential of integrated STEM education (Kelley and Knowles 2016).

A variety of useful conceptual frameworks for integrated STEM have been offered (e.g., see Asunda 2014; Asunda and Mativo 2016; Kelley and Knowles 2016; and English 2016). Moore and colleagues (e.g., Glancy et al. 2014; Guzey et al. 2016; Moore and Smith 2014; Moore et al. 2014) provide empirical support for the proposition that engineering is an essential bond or connector that can integrate STEM disciplines in K-12 education, as well as a facilitator of problem solving, creative thinking, communication and teamwork skills, and positive motivation and attitudes towards STEM careers. Engineering can be a motivator as a natural way to learn how to integrate STEM concepts, because real world engineering problems are often complex and require the application of mathematics and science. Ideally, this integration would go beyond the blending of “traditional types of understanding,” and therefore, “new models of instruction including curricular models must be developed if STEM integration is to lead to meaningful learning” (Moore et al. 2014, p. 41). For example, engineering design challenges are frequently taught through project-based learning (PBL) and collaborative learning activities. For many teachers, this would require adequate teacher PD, institutional structures that support integration, and integrated curricula (Glancy et al. 2014).

Perhaps most comprehensively, the National Academy of Engineering and National Research Council presented a framework for integrated STEM education in *STEM integration in K-12 education: Status, prospects, and an agenda for research* (Honey et al. 2014). The *framework* is comprised of four features: (1) goals, (2) outcomes, (3) nature and scope, and (4) implementation of integrated STEM education. *Goals of integrated STEM education* include STEM literacy, 21st century competencies, STEM workforce readiness, and interest and engagement of students in STEM subjects. *Outcomes of integrated STEM education* include outcomes for students such as learning and achievement and 21st century competencies and outcomes for teachers such as changes in practice, improved understanding of STEM content, and increased pedagogical content knowledge (PCK). Three important elements were identified that characterize the *nature and scope of integrated STEM education*: (1) type of connections among the STEM disciplines, (2) disciplinary emphasis or dominant discipline, and (3) duration, size, and complexity of the initiative (e.g., a single project, single course, curricular program, or entire school). The committee identified three important factors impacting *implementation of integrated STEM education*: instructional design, educator supports, and adjustments to the learning environment.

Conceptual frameworks such as these make increasingly clear that integrated STEM is not only about the STEM disciplines. It is frequently rooted in project- and problem-based learning, student-centered pedagogy, and 21st century transferrable skills. It promotes students as active learners; inventiveness, creativity, and critical thinking are fundamental aims.

Research has found that integrated STEM teaching encourages student-centered pedagogies (Roehrig et al.^2012^) and a more authentic treatment of mathematics and science content (Stohlmann et al. 2012). To some extent, STEM education (as with inclusive STEM schools) has focused on interdisciplinary, authentic, and contextualized problems (LaForce et al. 2016)—even before more contemporary movements towards NGSS and integrated STEM. What appears to be new is the serious pursuit of “STEM classes” and “STEM teachers”—i.e., classes that teach STEM subjects together in an integrated way, by “STEM teachers” rather than single-subject specialists. This possibility can lead to new “STEM” degrees, certifications, endorsements, and teacher PD, meaning specifically an integrated approach to teaching STEM. Indeed, this activity has already started (see Glancy et al. 2014). In keeping with common practice, the implied meaning of “STEM education” throughout this article is typically “integrated STEM education.”

### Evidence of STEM effectiveness

Many benefits have been associated with STEM education, such as providing opportunities for more student-centered, meaningful, engaging, and less fragmented learning experiences involving higher-level thinking and problem solving skills (Stohlmann et al. 2012). Students receiving education in STEM are thought to be capable of thinking logically and utilizing technology independently to solve problems, innovate, and invent. Integrating STEM disciplines has been associated with positive effects on attitudes in school (Bragow et al. 1995), achievement (Hurley 2001), and learning (Becker and Park 2011). Studies have also found that students exhibit higher levels of motivation and performance within STEM disciplines when engaged in activities such as prototyping, designing solutions, and utilizing technology like 3D printers (Tillman et al. 2014). This may be because integrated approaches lead to student perceptions of instruction being more relevant, active, challenging, meaningful, and competency-supportive, all perceptions related to greater student engagement (Shernoff 2013). Becker and Park’s (2011) meta-analysis of 28 studies on the effect of integrated STEM efforts on student learning reported mostly positive effects on student learning, with the largest effect sizes in elementary education and the smallest in college education. The meta-analysis demonstrated that integrated approaches can provide a rich context for interest development in addition to cognitive benefits.

The research on student outcomes has been regarded as inconclusive, however, especially from a long-term perspective (English 2016). For example, while a major hope for integrated STEM is the encouragement of engagement, motivation, and perseverance, these outcomes are rarely measured in evaluations of integrated STEM initiatives (Honey et al. 2014). Moreover, as English (2016) has pointed out, integrated STEM education is an embryonic field, and research to date raises more questions than it answers. From our perspective, chief among these questions is that of *feasibility.* So long as public schools continue to teach STEM, and indeed all subjects separately, challenges of implementing integrated STEM approaches in K-12 education promise to be significant. What is the realistic potential to implement integrated STEM education on a wide scale? Research suggests that teachers struggle to implement new and learner-centered paradigms of STEM education effectively even after demonstrating a deepened conceptual understanding through teacher professional development (Han et al. 2015). On a broader scale, recent STEM education reform efforts supporting inclusive STEM-focused schools, although introduced with great fanfare, have frequently dissolved due to a variety of contextual, policy, teacher, and student factors sorely underestimated (Weis et al. 2015). Integrated approaches to STEM education such as that propagated by the NGSS require a fundamentally different instructional approach in which the teacher assumes a facilitator role of student-directed and sustained investigations for challenges. The following are some of the related barriers that have been identified to advancing STEM education as an interdisciplinary study in K-12: (a) poor preparation and shortage in supply of qualified teachers, (b) lack of investment in teacher PD, (c) poor preparation and inspiration of students, (d) lack of connection with individual learners, (e) lack of support from the school system, (f) lack of research collaboration across STEM fields, (g) poor content preparation, (h) poor content delivery and methods of assessment, (i) poor conditions and facilities, and (j) lack of hands-on training for students (Ejiwale 2013). Most of these barriers go far beyond the challenges of conceptual integration among the STEM disciplines.

### Teachers’ content and pedagogical knowledge

Implementation of integrated STEM involves the inherent challenge of supporting a strong conceptual and foundational understanding of key concepts within multiple disciplines. Additionally, constructivist pedagogies including exploration and discovery may require teacher education both in educational foundations, science-focused principles, and pedagogical knowledge (Honey et al. 2014; Stohlmann et al. 2012). Pedagogical knowledge also plays a large role in teacher efficacy, which has been found to be very important for effective teaching (Stohlmann et al. 2012).

A central premise of this study is that it is important to give voice to the teachers and school administrators who actually have the needs and experience potential barriers in their ability to move towards integrated approaches in STEM. Although teachers are at the center of K-12 education’s expansion into integrated STEM approaches, many of the policies shaping K-12 education are formed with little to no input from teachers (National Academies of Sciences, Engineering, and Medicine 2017).

### The current study

In phase 1 of the study, we interviewed a limited, purposive sample of key teacher and administrator informants, asking them questions centered on primary research questions designed to inform a phase 2 statewide questionnaire study. Our research questions were as follows:

1. What are the greatest challenges to effectively implementing integrated approaches to STEM education? What supports would be most helpful to overcome these challenges?
2. What recommendations for pre-service education could help teachers better integrate STEM subjects? What recommendations for in-service or continuing professional development would help support integrated STEM education?

This second research question is not based on a supposition or opinion that integrated approaches to STEM are a direction that teacher education *should* take. Rather, it is based on the acknowledgement that delivering integrated projects, classes, and programs in K-12 schools is the present major agenda for STEM education of the Department of Education within the state examined—a direction heavily driven by industry and business for the perceived benefits towards desirable qualities of the work force. Given that K-12 schools will be increasingly called on to deliver integrated STEM, our research questions merely seek to identify teachers’ current needs and the supports that would need to be in place for this goal to be realized. This study was designed to illuminate the many factors that would need to be considered to enable teachers to be successful in integrating STEM subjects, including enabling skills and practices such as project-based and collaborative learning teaching methods. We intentionally left these questions open-ended, encouraging participants to expand on their answers in semi-structured interviews in order to inform item and response development in the phase 2 questionnaire.

## Methods

The present study utilized a qualitative, ground-up approach, relying on participants to articulate their own challenges and needs or perceived supports in STEM and integrated STEM education. Thus, patterns identified were rooted in participants’ *own descriptions.* We were committed to ascertaining the present challenges and needs of integrated STEM education as described by teachers and administrators. We then synthesized descriptions into coding categories for analysis.

### Participants and procedure

Participants were K-12 teachers (*n* = 22, including four “master teachers”) and administrators (*n* = 4, three principals and one math/science district supervisor) working in the state’s public schools. By focusing on teachers and administrators selected as leaders through a competitive grant process, we relied on *key informants* with a good understanding of challenges and needs in integrated STEM to maximize information from knowledgeable sources (Nagle and Gagnon 2008).

All participants in the key informant interview phase were also attendees of an integrated STEM Academy hosted by the state’s DoE in the summer of 2016. Attendance at the Academy was based on being a recipient of the state DoE’s competitive grant program in STEM education. The purpose of the grant-funded program was to support teachers who were interested in being leaders and affecting change in STEM education. Grant recipients received the 5-day summer academy and a limited amount of additional resources for the development of integrated STEM curricula and instruction upon request. Participants were nominated and supported by their school and/or district in the grant application process and were selected for inclusion by the state DoE. Approximately 67 teachers from 19 districts were grant recipients; all of these teachers attended the summer STEM Academy, along with an administrator from each district. Study participants were then selected at random from this larger group of STEM academy attendees.

In the STEM Academy, participants attended a variety of workshops on integrated STEM conceptualization, based on the state DoE’s definition of STEM education provided in the “Defining integrated STEM education” section above. Workshops were led by DoE officers, STEM experts, and five master teachers who had won either a Presidential Award for Excellence in Mathematics and Science Teaching or a Milken Educator Award and had significantly contributed to the development of STEM education. Because participants gained a shared understanding of integrated STEM from the workshop (e.g., the DoE’s definition of STEM education was provided to all participants), it was not further defined or conceptualized during the interview.

The research team obtained a list of STEM academy attendees from the state DoE and based phase 1 sample size on available time for four researchers to interview participants at designated times during the 5-day workshop. We randomly selected and interviewed as many of the master teachers and administrators as possible within time constraints^1^ due to their status as knowledgeable, key informants. Only those interviewed were inducted as study participants.

The final sample of 22 teachers represented a good balance of elementary, middle, and high school teachers and a good balance of science disciplines, technology/engineering, and mathematics. Eight of the 22 teachers taught in a high school (36%), eight taught in a middle school (36%), five taught in an elementary school (23%), and one worked at the district level (i.e., K-12; 5%). Eight were science teachers (36%), five were mathematics teachers (23%), four were technology teachers (18%), three taught other subjects (e.g., gifted/talented teachers or coordinators; 14%), and two were elementary school teachers without a specific subject (9%). Seventeen of the 22 teachers (77%), and two of the four (50%) administrators, were female.

### Data collection

We utilized semi-structured interviews as our primary data source. Interviews have several advantages in a needs assessment study: providing in-depth information on a broad range of topics, allowing for the explication of ambiguous responses, and eliciting more information than is possible through group methods. Interviews can serve as a useful starting point for the construction of questionnaires by identifying potential issues for further investigation (Nagle and Gagnon 2008).

The interviews ranged from 25 to 45 min in length. Four experienced researchers conducted the interviews. We utilized slightly different interview protocols for teachers and administrators. The teacher interview began by asking participants their greatest needs and challenges *within their teaching area or subject* and what supports would best help them to overcome their challenges. This established a *baseline* *of teacher needs* as a point of contrast to the needs and perceived supports enabling integrated STEM approaches *beyond their ordinary instructional needs*. Next, we asked for the extent to which they integrated multiple disciplines in their instruction. Remaining questions were as follows: What are the *barriers or challenges* to achieving integrated STEM education? What *supports* would you need to do more with integrated approaches to STEM, or to take it to the next level? How would you envision *teacher education* that would be most helpful to develop adequate qualifications as a STEM teacher? In terms of *pre-service education* and preparation? In terms of *continuing professional development and in-service*?

We also interviewed several administrators (*n* = 4) about integrated STEM activities in their schools or districts; their greatest challenges and needs related to achieving integrated STEM education; the most desired supports, resources, and staffing needs to teach STEM in an integrated way; the qualifications of ideal STEM teacher candidates; and suggestions for teacher education and PD.

### Data analysis

The team that conducted the interviews analyzed the data to determine themes, as has been suggested for qualitative data analysis (Creswell 2007; Denzin and Lincoln 2005). Recorded interviews were divided randomly among the four researcher-coders. Using a grounded theory approach, we developed a systematic and organized process for the emergence of themes (Glaser and Strauss 1967). An initial step was identifying essential elements, by conceptualizing, examining, and categorizing the data. All coders generated categories and discussed them. This led to selective coding in which we determined essential and core categories, consolidating some codes into central and comprehensive codes. Preliminary codes were often idiosyncratic to a single interview. For example, one respondent identified “student-centered learning” as something that was needed to take STEM to the next level, and this was the code initially given. However, she elaborated that what needed to be in place was a school ethos that valued student-centered learning. When viewed in light of several other responses discussing the theme of a “supportive STEM ethos or culture,” this latter code was deemed the more comprehensive “theme” running throughout the interviews. Final codes can be found in Table 1. We allowed for double coding, meaning that more than one topic could be identified as being discussed by one interviewee. Twenty-five percent of the manuscripts were coded by two coders to estimate inter-rater reliability. Inter-rater agreement was 90%, and Cohen’s Kappa (which takes into account the rate of random agreement) was .80, which is in the acceptable to good range.

**Table 1:** Coding categories and frequencies of participants discussing each

**Present integrated STEM instructional activities**	**Supports needed to advance integrated STEM**
• Integrated a combination of some subjects (14)	• Time to collaborate and plan (8)
• Integration of technology (11)	• Teacher professional development (7)
• Application to real life problems (8)	• Resources (6)
• Already doing integrated STEM classes/programs (4)	• Supportive STEM ethos/culture (4)
• Minimal or no integration (4)	• Communication between departments (4)
• Integration of student-driven labs (1)	• More instructional time (3)
	• Shift in teacher attitudes (1)
**Challenges to achieving integrated STEM**	• More manageable class sizes (1)
• Lack of teachers understanding (8)	
• Lack of time for collaboration and planning (8)	**How to improve pre-service education**
• Barriers of school organization (6)	• Integrated multidisciplinary approach (9)
• Lack of instructional time (5)	• Observe effective STEM lessons (5)
• Testing culture as competing value (5)	• More classroom experience (4)
• Lack of understanding disciplinary content and standards (5)	• Good teaching mentor (3)
• Teacher resistance (4)	• More rigorous science/math training (3)
• Lack of technology (4)	• Better understanding of NGSS (2)
• Lack of finances (3)	• Experience writing objectives (1)
• Lack of resources (3)	• Learn how to reduce direct instruction (1)
• Lack of administrative support (1)	
• Lack of time for planning (1)	**How to improve in-service education**
• Lack of professional development (1)	• More integrated STEM training (7)
• Lack of student motivation (1)	• See examples of effective lessons (7)
• No barriers (1)	• Talk to/observe out-of-district teachers (3)
	• Rely on expertise of district teachers (2)
	• Create a supportive community (2)
	• Require follow-up after trainings (2)
	• More time to for teacher collaboration (2)
	• More individualized lesson plans (1)
	• Review and use emerging technologies (1)
	• Discipline-specific training (1)

## Results

Results are discussed by topic, focusing on the categories that were mentioned most frequently. Table 1 provides the frequency with which each theme was discussed. Because we allowed for double coding, each frequency count is independent from the others. Results were analyzed by elementary, middle, and high school levels; notable differences are reported where applicable.

### What were teachers’ greatest needs and desired supports in their own teaching area?

We first asked teachers to identify their greatest teaching needs and challenges within their teaching area to establish “baseline” needs. Responses are summarized below (not shown in Table 1). Overall, the most frequently discussed topics were (a) lack of physical resources and technology (7 of 22, or 32%), (b) dealing with changes in student expectations and attitudes (6 of 22, or 27%), (c) dealing with mixed abilities and gaps in students’ understandings (5 of 22, or 23%), (d) lack of student interest and engagement (4 of 22, or 18%), (e) lack of time for planning and collaboration (3 of 22, or 14%), (f) completing paperwork (3 of 22, or 14%), and (g) lack of district administrative support (3 of 22, or 14%). When asked what supports would help to overcome their challenges or become a more effective instructor, the most common topics were (a) more resources and technology support (9 of 22, or 41%), (b) support for collaborating and planning with teacher colleagues (8 or 22; 36%), (c) more professional development (6 of 22, or 27%), (d) deeper backgrounds and greater interests in topics among students (2 of 22, or 9%), and (e) new standards focusing on fewer topics more deeply (2 of 22, or 9%).

### How had teacher participants integrated STEM disciplines in their instruction to date?

Participants were asked, “Have you integrated multiple disciplines such as science, math, and/or engineering in your instruction?” Themes and frequencies of responses are shown in Table 1. The majority of teachers (14 of 22, or 64%) reported that they had integrated a combination of some subjects. Several reported that they were already implementing integrated STEM education. Now existing in some districts, according to several administrators, were “integrated” science classes to support new science standards (i.e., NGSS) and (Common Core) mathematics standards, which encourage attention to application. In some schools, STEM existed as a special arts class. Nevertheless, four teacher participants (3 of 8 high school teachers, 1 of 8 middle school teachers, and no elementary school teachers) reported that integrated STEM activities were minimal or did not yet exist.

Half of the teachers (11 of 22; 50%) indicated that they integrate technology into their lessons. Of those who reported technology integration, some identified fairly standard classroom technologies, such as smart boards and laptops, while others reported more innovative technology integration such as student creation of digital books and playing interactive educational video games. Approximately one-third of teachers (8 or 22; 36%) reported that they tried to integrate STEM by drawing on real-life problems.

Elementary teachers were more likely to believe that they were already integrating STEM subjects, seemingly because separate subject classes are not as commonplace, especially in the early grades. In middle and high school, the extent of integration was mixed, but many teachers had experimented with integration in some meaningful way, with some projects involving coordination across subjects. For example, one mathematics teacher conducted an integrated project on copper-plating. Data were collected in science class and analyzed in math class. An educational technology instructor implemented units integrating science and mathematics, such as building a birdhouse. One science teacher developed an activity to build bridges, integrating geometry to solve problems such as how much weight it can hold. Another unit used Play-doh to model the solar system; students estimated size, scale, and distance between planets. Some teachers were rather sophisticated with their integration. For example, a vocational technology teacher developed several integrated units including one to design and construct buildings with wood supplies. Other units centered around biotechnology, transportation, medical tools, hydroponics, and agriculture, with the goal of students’ obtaining strong connections to viable vocations.

Our master teachers already had career trajectories demonstrating commitment to curricula and instruction integrating STEM disciplines. One master teacher was one of two teachers teaching in a STEM “school within a school.” What had started as a special STEM program became recognized as educationally superior and grew in popularity and size. For her, nearly all of her curricula and instruction was integrated. Another master teacher with an engineering background and graduate degree had developed a variety of integrated STEM activities centering around the engineering design process, not dissimilar from those demonstrated in the current literature (e.g., Estapa and Tank 2017; Glancy et al. 2014; Grubbs and Strimel 2015; Guzey et al. 2016). This teacher shared her work catalyzing change in a STEM academy high school to engage students in the engineering design process and “school-based inquiry” in which students use engineering design to solve various problems. She facilitated a variety of school-wide activities, from First Lego Leagues to participating in a NASA-sponsored competition to design rockets (involving video conferences with NASA engineers and astronauts), to STEM expositions for student exhibits of STEM projects.

Another master teacher had a background in science and medicine, started teaching as a biology teacher, and currently serves as a high school and university adjunct instructor in programs that promote health-related careers. In her anatomy and physiology course, students researched the causes and therapies of a variety of skeletal and muscular diseases and traumas, including injuries requiring amputation, diabetes, and traumatic burns. After this initial research phase, her students worked in mixed-ability groups to design a prosthetic hand with the ability to pick up, hold, and release. Students needed to consider a variety of facts and concepts, from the angles of bends to the various tendons and ligaments connecting muscles and bones. In another activity based on the medical profession, each student would play the role of doctor, patient, or medical transcriptionist. Doctors needed to elicit information from the patient such as symptoms and medical history, before running tests. Science and mathematical knowledge and skill was required especially in diagnosing various ailments. Connections to college preparation and health-related careers were made throughout these courses.

### What were the challenges to achieving interdisciplinary STEM instruction?

Teachers were asked, “What are the barriers or challenges to achieving interdisciplinary STEM education?” Responses to this question represented a range of 15 different coding categories. Among the most frequent challenges identified was a lack of understanding (8 of 22, or 36%). Many teachers explained that they simply did not know how to effectively integrate the STEM areas, although this was not identified as a challenge among elementary school teachers. Some teachers explained that their lack of understanding of how to teach in integrated ways was strongly related to students’ lack of understanding or lack of motivation to learn in different ways. They asserted that a shift in mindset was needed on the part of both students and teachers in order to understand that the teacher’s role was not to give students the correct answer. Administrators provided a somewhat different but not incompatible perspective. They had a realistic sense of how deep the challenges for teacher education in integrated STEM run, owing to the fact that most teachers went to college before integrated STEM was conceived. They recognized that individuals who truly have a firm conceptual framework in integrated STEM approaches are scarce commodities.

Related to this barrier, multiple participants also expressed the need to develop an understanding of content and standards in subjects that they do not teach. As one 6th grade mathematics teacher participant said, “I’m good with the hands-on things and putting things together and figuring things out; (with) that I’m fine. But when it comes to (the) actual science part, like why it works and why some things are getting done, the standards of science are my downfall. It’s like I’m looking at a different language. I’m like, ‘what does this mean?’” Relatedly, some teachers observed that there appeared to be multiple understandings about what engineering instruction is or could be and desired this to be clarified.

Another challenge commonly discussed was lack of time—both for collaborative planning (8 of 22, or 36%) and for integrated STEM instruction (5 of 22, or 23%). Teachers reported that there is a lack of time to work with fellow teachers to develop multidisciplinary curriculum activities. According to interviewees, such time together would enable teachers to generate ideas and piggyback on each other’s work. Time for collaboration would foster the teamwork necessary to support integrated STEM education. In terms of instructional time, a challenge discussed mostly by high school teachers was that STEM projects require longer periods of time, making lack of sufficient instructional time a serious challenge. Several suggested that STEM should be its own class.

Another barrier, reported by at least 20% of the teachers, included issues related to school organization and structure. Participants acknowledged that the schedule of classes during the school day restricted possibilities for integrating STEM in their classrooms. Middle and high school teachers in particular reported limitations in their ability to find common blocks of time that would allow teachers of multiple STEM disciplines to collaborate, develop, and implement integrated lessons. They also reported that with so much content to cover, there is not enough instructional time for STEM projects. Elementary school teachers, none of whom reported school organization as a challenge, indicated that they had greater flexibility during their day to devote significant blocks of time to working on integrated STEM lessons.

An additional concern voiced by multiple teachers (5 of 22, or 23%) was the impact of testing. This was a pertinent problem especially at the high school level. These teachers stated that there is substantial pressure to ensure students are ready for standardized tests and that test preparation takes away from instructional time for creative and integrated forms of instruction such as problem-based or project-based learning. Meanwhile, administrators discussed the need for changes in how to measure learning or achievement in integrated STEM, especially with respect to the essential “soft skills” or non-cognitive factors like persistence in the face of failure.

Several teachers also identified insufficient resources and instructional material (3 of 22, or 14%) and a shortage of finances (3 of 22, or 14%) as important challenges. It is worth noting that the lack of resources was the most frequent challenge discussed within teachers’ own disciplines, mentioned by half of the teachers interviewed, before even considering additional resources and instructional materials that STEM projects would require.

### What supports were needed for more integrated approaches to STEM?

Teachers were asked, “What supports would you need to do more with integrated approaches to STEM or to take it to the next level?” Consistent with previous responses, the most frequent support mentioned (8 of 22, or 36%) was time to collaborate and plan. In order to take integrated STEM education to the next level, teachers reported that they need more time for different content areas teachers to work together. More instructional time was identified as a desired support by several teachers, all of whom taught in high schools.

Second, many teachers (7 of 22, or 32%) discussed the need for more teacher PD. They indicated that in order to be effective, they needed additional PD on how to incorporate and connect different STEM disciplines and on technology integration. Administrators saw the financing of sufficient PD as among the greatest needs to achieving their goals, especially for STEM curriculum development and instructional implementation. Several administrators were insightful regarding the shift in mindset needed to achieve STEM integration, believing that it is important, for example, for teachers to understand technology use in the context of the engineering design process rather than more superficially characterizing technology incorporation as any type of student computer use. Some administrators recognized the important role of PD in reinforcing STEM conceptual framing, including evidence of its effectiveness. For example, one administrator believed that a key function of PD could be in leveraging the engineering design process and project-based learning as guiding philosophies. Some suggested that on-site PD would be the most helpful, in order for the PD to be more customized to the uniqueness of the school environments and curricula.

Relatedly, we asked administrators if they currently wanted to hire a STEM teacher. While most expressed an interest in this, our administrators recognized the lack of a bona fide pool of candidates, owing to the current shortage of truly integrated STEM pre-service teacher education programs. The administrators believed that the ideal STEM teacher would not be intimidated by mathematics and science, but rather would have a passion for and a solid content expertise in both—along with proficiency in related disciplines. Good candidates would also have a comfort level with exploring uncharted territory, trying new things, adapting, and the inevitable failure that comes with experimentation.

Multiple interviewees (6 of 22, or 27%) reported that additional resources would help them take integrated STEM to the next level. Having good exemplars, models, structured lesson plans, and materials in integrated approaches were all identified as important needs for achieving STEM integration. Such resources also included materials, equipment, movable furniture, and other physical resources to create learning environments conducive for science inquiry. Administrators also understood how important resources are to achieving STEM goals.

Several teachers, mostly from middle schools, discussed the need for communication among all departments to facilitate cross-curricular initiatives. Finally, some administrators discussed the importance of tapping into community supports and resources, including parents.

Although it was not a question on our interview protocol, several teachers spontaneously described some supports for integrated STEM activities that they currently have. Three teachers discussed ways in which their current math/science supervisor was very supportive of integrated STEM activity. Two teachers noted that the common planning time that is already provided (e.g., twice per week) has been very helpful in their planning of integrated STEM lessons.

### Recommendations for pre-service education

Participating teachers reported that their own pre-service preparation emphasized pedagogical practices, content area knowledge, and curricular standards. While this helped them to improve the quality of teaching in their content area, it was inadequate for current efforts towards integrated STEM instruction. To overcome the challenge of knowing content and standards in subjects in which they had not specialized, many teachers (9 of 22, or 41%) suggested a redesign of pre-service programs in order to nurture integrated approaches explicitly. They suggested inclusion of coursework that focused on learning standards in all of the STEM subjects and pedagogical practices that support STEM integration such as project-based and collaborative learning activities. Some teachers further proposed that pre-service education should include courses on theoretical foundations of cross-curricular and multidisciplinary approaches for STEM learning.

Other suggestions for pre-service education included the availability of model or exemplar STEM lessons (7 of 22, or 32%) and mentoring by teachers who were experienced in integrated approaches (3 of 22, or 14%). Teachers expressed the desire to observe experienced STEM teachers in action in order to see what integrated STEM lessons “look like.” Some respondents asserted that due to the predominant testing culture, many teachers do not intuitively understand project-based learning. Teachers suggested that classroom observations were also needed to provide guidance on how to manage STEM classrooms. Consistent with the discussion of challenges and supports, some teachers believed that pre-service education needed to emphasize student-centered pedagogical approaches, where learning by experience and asking rather than answering questions is valued. Some teachers suggested that additional teaching credentials, such as certification, endorsements, and certificates would support the additional education needed.

Administrators also recognized the need for new pre-service education programs in integrated STEM. They suggested that they would like to see new pre-service programs provide experience with science and engineering practices; education on real world applications, play, and creativity; and the linking of instruction to jobs and careers in order to establish relevance. They believed that high-quality programs would also help to develop expertise in science, math, and engineering standards and how to effectively engage students in projects meeting those standards.

No teachers asserted that changes to pre-service education were not important or needed, or that other kinds of changes to education were more important. However, we do not know the extent of any response bias caused by the suggestiveness of the interview question itself. We also do not know what teachers might have said if we had asked them to evaluate the relative importance of changes to pre-service education supporting integrated STEM directly.

### Recommendations for in-service professional development

Teacher participants were past their pre-service education, but they still participate regularly in continuing in-service PD. Similar to their suggestions for pre-service education, many teachers (7 of 22, or 32%) suggested that more in-services were needed to help them with integrated approaches to STEM education explicitly. Approximately one-third of the participants (7 of 22, or 32%) reported that it would be helpful if they had the opportunity to review exemplar units in key pedagogical strategies such as project-based learning, a suggestion echoed by administrators. Both teachers and administrators suggested the importance of opportunities to see video recordings of experienced teachers implementing integrated STEM lessons and to access exemplar lesson plans or units. It was also suggested that PD days be devoted to helping teachers experience problem solving using multiple STEM disciplines. For example, one teacher desired the opportunity to experience the engineering design process first hand—first as a learner, followed by developing a higher level of mastery to approach it as a teacher.

Administrators suggested that including mathematics, science, and engineering teachers together in PD would be very important for meeting the goals of integrated STEM. Some stressed the importance of emphasizing the integrated STEM “vision” to create buy in and to generate enthusiasm about the immense potential that exists if teachers worked together on more ambitious projects than would be possible working separately. Forging and sustaining collaboration between teachers within schools and across districts was also suggested as a valuable strategy. Meanwhile, some teachers suggested that administrators needed to conduct more classroom observations to make informed policy decisions related to integrated STEM. Teachers looked forward to opportunities where they could be given time to share, discuss, and receive feedback on strategies for effective STEM integration with fellow colleagues both within and across school buildings.

## Discussion

In this article, we discussed phase 1 of a two-phase, professional development needs assessment study in integrated approaches to STEM education. The interviews conducted in this phase of the study were intended to inform construction of a questionnaire, currently in progress, to be administered statewide. We seek to understand the greatest perceived challenges and needs of teachers and administrators throughout the state related to teaching STEM subjects in an integrated way and what supports would be the most welcomed in the effort to move towards more instructional integration in order to inform resource and policy decisions. Findings and conclusions at this point in the study must therefore be regarded as preliminary.

Our interviews yielded several distinct themes. Overall, they suggested that integrated STEM activity is occurring in small pockets but is not yet pervasive. The master teachers that we interviewed provided compelling models of teachers engaging in integrated STEM education. However, it was apparent that most teachers lagged far behind the vision and promise for integrated STEM that the master teachers demonstrated was possible. The teachers in our study recognized that their inability to envision what teaching integrated STEM “looks like” was their greatest current obstacle. As Moore et al. (2014) observed, many teachers have gaps in the content knowledge of their own subjects and expecting teachers to be familiar with other subjects creates more gaps. Some teachers readily admitted that they lacked sufficient knowledge of content and standards of the STEM subjects other than the one that they teach. Because an integrated approach focuses on big ideas that are connected and interrelated, support is needed to help teachers with instructional approaches that organize knowledge around big ideas, concepts, and themes (Stohlmann et al. 2012).

Teachers wanted to participate in PD that was taught using integrated STEM approaches, so that they could *experience,* from the perspective of a student, good exemplars. Broadly speaking, the teachers that we interviewed had a sense of how to begin working towards an integrated STEM approach, but realized that they needed help: more collaboration, more modeling, more exemplars, and more mentoring.

### Needs, challenges, and desired supports unique to integrated STEM

Beyond lack of knowledge and vision, teachers mentioned a number of other challenges, not all of which are completely new but may be particularly meaningful when applied to integrated STEM instruction. These include needs related to lack of time (both for collaborative planning and instructional time for integrated STEM lessons), school structure and organization, state testing, assessments for STEM achievement, perceived lack of resources, and teacher education (both pre-service and continuing in-service PD), and visible models of STEM integration.

#### Perceived lack of time

Lack of time was among the most frequent need mentioned—both time for collaborative preparation and instruction. Teachers are notoriously pressed for time, and so this is far from new as a teacher challenge. In fact, approximately a third of teachers interviewed noted that collaborative planning time was among their greatest needs for effective instruction in their own subject, without regard to cross-department collaborative planning supporting integrated STEM instruction. Even more teachers identified this as a challenge when discussing needs for integrated STEM specifically, however, especially among high school and middle school teachers. The need for instructional time for integrated STEM activities or lessons, mentioned most frequently by high school teachers, was not discussed as a challenge within teacher’s disciplines at all.

#### Time for collaborative planning

There were aspects to teachers’ challenges around time that were unique to the demands of integrated STEM. Teachers expressed both the desire and need for time to *collaborate* with other instructors. Until more teachers have more education in disciplines outside of their teaching area, or in STEM specifically, successful STEM integration is largely *dependent* on collaboration among teachers from multiple disciplines. Key informants were highly aware that integrating STEM would not be achieved without more time to work together. Teachers believed that collaborating with other teachers from different disciplines was essential to improve their understanding of other subject areas and to develop multidisciplinary, integrated approaches.

#### Instructional time

There are ways in which the need for more instructional time is also uniquely related to the integrated STEM approach. Project-based and discovery-based approaches take considerably more time than does direct instruction. Attempting to reach learning goals from multiple content areas in the context of such projects, as described by Moore et al. (2014), can also take appreciable amounts of time to implement. The suggestion by many high school teachers that STEM needs to be its own class certainly underscores the need to incorporate additional instructional time for it.

#### School structure and organization

Relatedly, teacher participants acknowledged that the school schedule heavily impacted possibilities for collaborative planning time as well as instructional time for STEM integration. Middle and high school teachers in particular reported limitations in terms of finding common blocks of time that would allow teachers of multiple STEM disciplines to collaborate and deliver integrated lessons. If collaboration and instructional time are essential requirements due to the interdisciplinary nature of the desired changes, schools may need to seriously reconsider issues of the school schedule and how the various goals of schooling are organized.

#### State testing

Another structural challenge relates to the challenges of testing (e.g., PARCC, Common Core testing), a concern expressed by high school teachers in particular. While teacher challenges over testing and the highly related issues of content coverage are already a widely discussed and debated topic, in this study it was not a notable topic of discussion when teachers were asked about challenges within their own discipline. This does not mean that our participants never experience challenges related to testing in their own subject. A reasonable interpretation, however, is that teachers have difficulty imagining effectively instituting cross-disciplinary STEM instruction while the accountability for within-subject content mastery remains intense. Whereas testing has been widely reported to diminish time for creative and innovative instructional approaches that many teachers *desired,* testing diminishes time for the pedagogical approaches that STEM instruction *requires*. Indeed, the goals of content testing are experienced by teachers as diametrically opposed to the goals of integrated STEM education. Thus, one important consideration for policy makers and administrators is clarity around any trade-offs for taking on a radically different instructional approach. Teachers appear to recognize that they cannot simply add integrated STEM on top of their current expectations. Rather, their responsibilities would need to change in clearly defined ways.

#### Assessments for STEM achievement

Because state testing influences content taught in classroom, appropriate methods of assessment for integrated STEM curricula also promise to be another important area that needs to be reconsidered and rethought. Policy makers and administrators will need to consider an appropriate response to changes in instructional expectations with respect to standardized testing. Alternative assessments that evaluate students’ application of concepts in addition to knowledge and comprehension will be needed. As mentioned by some of the school administrators, schools will also need to consider how to measure achievement with respect to “soft” or non-cognitive skills like persistence and teamwork.

#### Perceived lack of resources

A perceived lack of resources is also not a new problem; indeed, it was the most frequent challenge discussed within participants’ teaching areas. However, materials for integrated STEM activities undoubtedly add an additional layer of materials that would be needed, especially engineering supplies and software, as well as materials and tools for building and “making,” such as wood, plastic, and machinery. These sorts of resources were more likely to be needed at the high school level. Our findings suggest that lack of adequate resources is something that policy makers and administrators need to consider because the expectation of integrated STEM approaches requires these kinds of materials and tools.

#### Teacher education

Perhaps the most salient preliminary finding from this phase 1 study is the nearly universal belief that current teacher education is wholly inadequate and would need to be considerably rethought and revamped, for integrated STEM to flourish in schools. Teachers’ responses suggested the need for STEM teacher education courses and workshops (both pre-service and in-service) to address the integration piece directly by demonstrating how the multiple STEM disciplines can be bridged through teamwork in real-world problem solving. An authentically integrated approach would emphasize the comprehensive and trans-disciplinary nature of scientific inquiry to form explanations, which in turn, may inform engineering design and technology use to solve problems. With respect to pedagogical practice, one teacher posited that teachers have come to rely on correct solutions just as students have and must become more comfortable with the inherent risks and potential failures that accompany project-based approaches. These unique challenges suggest a two-fold priority for teacher education programs in STEM. The first is to strengthen teachers’ content area knowledge and classroom management skills needed to efficiently run integrated STEM lessons. The second is to prepare teachers with pedagogical practices that encourage students to collaborate, deal productively with failure, and persevere. Therefore, teacher education needed for STEM appears to entail a complete redesign of the teaching and learning process—one that, at least initially, may require interdisciplinary teams of educators from multiple STEM disciplines. One interesting future direction is the extent to which future STEM teachers will need expertise in the standards and key concepts of all other STEM subjects, or whether there will be some consolidated set of principles considered most important for the STEM teacher of the future to master.

#### Pre-service education

Providing opportunities to experience interdisciplinary teams working together can be expected to be a challenging goal for pre-service education programs. Even sub-disciplines within science (i.e., biology, physics, and chemistry) are frequently seen as only marginally related to each other, and the various STEM disciplines still exist in different buildings in many institutions of higher education delivering pre-service education. Although there are some isolated teacher education programs in integrated STEM programs (see, for example, Glancy et al. 2014), we need to know more about how this can be realistically achieved on a wider scale.

#### In-service education

The nature of continuing professional development required to support teachers is another highly unique need of the integrated STEM movement. In particular, teachers attested to the need for continuing PD for certified teachers to focus on content areas and standards outside of teachers’ specialization and how to integrate multiple learning goals from different content areas. As suggested by Moore et al. 2014, engineering design challenges and other projects can be complex and involve both science and mathematics, but an essential ingredient is the skill of the teacher to facilitate conversations embracing this complexity, highlighting crosscutting concepts and aspects of science and engineering practices that unify the many “parts.” Teacher skill is also needed to promote teamwork and communication skills. Just like the PD required to support the engineering design and project-based models as discussed by Glancy et al. (2014), our interviews suggested that PD supporting integrated STEM must include constructivist, inquiry-based, and project-based pedagogy.

#### Models of STEM integration

Teachers repeatedly expressed the need to see examples of other teachers implementing integrated STEM lessons. By observing sample lessons, teachers see how curricula, instructional approach, and their interactions play out. Seeing rich examples of classroom interactions to effectively structure teachers’ sensemaking, including both video and live observations, has frequently been identified in the literature on best practices in teacher professional development (Ball et al. 2009; Borko et al. 2008; Roth et al. 2011; Sherin and Han 2004). Teachers welcomed opportunities to see models of lessons and appropriate assessments based on engineering design challenges. The effectiveness of such approaches in fostering student motivation and engagement as well as disciplinary content learning has been supported by a number of research studies (e.g., Estapa and Tank 2017; Glancy et al. 2014; Grubbs and Strimel 2015; Guzey et al. 2016). A variety of relationships between engineering and science may emerge as models become increasingly available.

### Implications for policy and decision makers

This study, especially the full 2-phase study, is likely to have implications for policy with respect to teacher certification, endorsements, and credentialing, including the possibility of alternative credentialing. State endorsement and/or certification requirements can drive new teacher certification programs offered by institutions of higher education. With many certified teachers feeling ill-equipped to implement integrated STEM, and recognizing needed shifts in the mindsets and practices of teachers and students that it entails, educational opportunities that are more substantial than isolated in-service workshops appear to be needed. So as not to reproduce the requirements and investments of a pre-service education for teacher certification, however, alternative credentials or “stackable” micro-credentials may be desirable, allowing teachers to flexibly apply STEM in various areas of specialization.

In a very real sense, the primary challenge schools face in teaching STEM subjects in an integrated way is the most unique of all to the integrated STEM movement: schools continue to teach STEM subjects separately. This can be considered a superordinate challenge subsuming most of the others, since many of the various challenges and obstacles identified by participants were strongly related to the separate teaching of school subjects in schools. There may be little time in school schedules for planning and teaching integrated STEM until substantive changes in school organization and scheduling are made. As Moore and Smith (2014) state, “School change is needed to support STEM integration. Schools are set up to silo the STEM disciplines…Schools need to make structural changes that will allow students to…learn the nature of each of the STEM disciplines and learn that they are interconnected in ways that…they will encounter in real world problems” (p. 7). The extent to which administrators and teachers understand and expect meaningful reform in how the STEM subjects (if not all school subjects) are presently taught is an important issue as we continue to study the needs and challenges associated with K-12 education in integrated STEM.

### Implications for phase 2 questionnaire construction

At a 2016 summer STEM Academy, our study participants received a definition of STEM from the state DoE that reflected a conceptual framework for integrated STEM education. However, teachers throughout the state cannot be expected to have an understanding or working definition of what integrated STEM is, let alone a common understanding. Definitions of integrated STEM education appear to vary significantly. Therefore, more needs to be known regarding teachers’ present understanding of integrated STEM and how it is presently conceptualized. Understandings of the basic components of integrated STEM are likely to range. For example, while many recognize the importance of integrating technology as the “T” in “STEM,” technology integration appears to be conceived in very different ways, reflecting the range of technology integration from superficial to transformative (Romrell et al. 2014). There were also suggestions in our interviews that understandings of engineering and engineering education vary as well. The phase 1 study also strongly suggests the importance of customizing questions to elementary, middle, and high school levels since teachers’ needs at those levels appear to be quite different. This is especially true given the tendency for elementary teachers to believe that they are already integrating STEM subjects. Although completion of the phase 2 questionnaire is beyond of the scope of phase 1, these observations will help to inform its construction.

### Limitations

The key informants in our purposive sample were chosen because they were nominated by their supervisors as teachers who have the potential to lead the integrated STEM movement in their districts and who welcomed further instructional and curricular support. It is important to bear in mind that both the selective sample as well as the shared experience of study participants in the STEM academy were likely to have biased the results. First, there was likely a selection bias toward more advanced teachers. Second, the level of acceptance and receptivity among the teachers towards integrated STEM as a desirable goal was likely higher than that of a random sample of teachers teaching STEM subjects. This likely resulted in response bias compared to a larger population of teachers. It is our intention to overcome this limitation in phase 2 of the study; however, it remains a factor preventing broader generalizability in phase 1.

In addition, responses to the kinds of questions asked in this study were likely to be influenced by the implementation stages of the teachers. For example, teachers are accountable for implementing NGSS in our state currently and we have observed a high level of stress and anxiety among some teachers in our providing of NGSS PD. In this study, implementation was not yet required, and therefore, most teachers’ dispositions towards integrated STEM can best be described as exploratory (with the exception of the master teachers). However, there was also variability in stage of implementation that was not measured.

## Conclusions

Given the growing interest in, and relevance of, integrated approaches to STEM education, there is an urgent desire to understand the challenges and obstacles in developing and implementing integrated STEM curricula and instruction. The preliminary findings in phase 1 of this two-phase needs assessment study provide a starting point for better understanding teacher needs in integrated STEM education. We discussed themes that were identified by at least several of our 22 teacher participants and were discussed in some depth during the interviews. Phase 2 of the study will allow us to consider new questions and directions arising from phase 1 findings in addition to those suggested by the literature.

The strength of the full two-phase study is in representing the on-the-ground challenges and supports necessary among those who would work most directly to implement integrated STEM curricula and programs of instruction for K-12 education. This is of critical importance given the significant variation across individuals, schools, and disciplines with respect to current understandings of integrated STEM education and its core components. The themes identified and discussed in phase 1 of this study help to provide a common language and springboard for further communication and research exploring the development of integrated approaches to STEM education in K-12 schools.

## Abbreviations

STEM: Science, technology, engineering, and mathematics

## Footnotes

1 Given the perceived importance and timeliness of the two-phase study results to the state, the research team and state Department of Education preferred not to have an extended period of data collection for phase 1 of the study. Instead, a wider representation of stakeholders will be utilized in piloting the phase 2 questionnaire.

## References

[CR1] Asunda PA (2014). A conceptual framework for STEM integration into the curriculum through career and technical education. Journal of STEM Teacher Education.

[CR2] Asunda PA, Mativo J (2016). Integrated STEM: A new primer for teaching technology education. Technology and Engineering Teacher.

[CR3] Ball DL, Sleep L, Boerst TA, Bass H (2009). Combining the development of practice and the practice of development in teacher education. The Elementary School Journal.

[CR4] Becker K, Park K (2011). Effects of integrative approaches among science, technology, engineering, and mathematics (STEM) subjects on students’ learning: A preliminary meta-analysis. Journal of STEM Education.

[CR5] Borko H, Jacobs J, Eiteljorg E, Pittman M (2008). Video as a tool for fostering productive discussions in mathematics professional development. Teaching and Teacher Education.

[CR6] Bragow D, Gragow KA, Smith E (1995). Back to the future: Toward curriculum integration. Middle School Journal.

[CR7] Burrows A, Slater T (2015). A proposed integrated STEM framework for contemporary teacher preparation. Teacher Education and Practice.

[CR8] Charette, R. N. (2013). *The STEM crisis is a myth.* Accessed 20 Apr 2017 from http://spectrum.ieee.org/at-work/education/the-stem-crisis-is-a-myth.

[CR9] Creswell JW (2007). Qualitative inquiry and research design: Choosing among five approaches.

[CR10] Dayton Regional STEM Center. (2011). STEM education quality framework. http://daytonregionalstemcenter.org/wp-content/uploads/2012/07/Final-Framework-copyrighted.pdf. Accessed 22 March 2017

[CR11] Denzin NK, Lincoln YS (2005). The Sage handbook of qualitative research.

[CR12] Ejiwale J (2013). Barriers to successful implementation of STEM education. Journal of Education and Learning.

[CR13] English LD (2016). STEM education K-12: Perspectives on integration. International Journal of STEM Education.

[CR14] Estapa AT, Tank KM (2017). Supporting integrated STEM in the elementary classroom: a professional development approach centered on an engineering design challenge. International Journal of STEM education.

[CR15] Georgetown University Center on Education and the Workforce (no date). *Science, technology, engineering, mathematics: STEM executive summary.* Accessed 20 Apr 2017 from https://cew.georgetown.edu/wp-content/uploads/2014/11/stem-execsum.pdf.

[CR16] Glancy, A., Moore, T., Guzey, S., Mathis, C., Tank, K., & Siverling, E. (2014). *Examination of integrated STEM curricula as a means toward quality K-12 engineering education.* Proceedings of the 2014 American Society of Engineering Education Annual Conference and Exposition. Indianapolis, IN, June 15th - 18th. Washington, D.C.: ASEE.

[CR17] Glaser B, Strauss A (1967). The discovery of grounded theory.

[CR18] Grubbs M, Strimel G (2015). Engineering design: The great integrator. Journal of STEM Teacher Education.

[CR19] Guzey SS, Moore TJ, Harwell M (2016). Building up STEM: An analysis of teacher-developed engineering design-based STEM integration curricular materials. Journal of Pre-College Engineering Education Research (J-PEER).

[CR20] Han S, Yalvac B, Capraro MM, Capraro RM (2015). In-service teachers' implementation and understanding of STEM project based learning. Eurasia Journal of Mathematics Science and Technology Education.

[CR21] Honey M, Pearson G, Schweingruber A (2014). STEM integration in K-12 education: Status, prospects, and an agenda for research.

[CR22] Hurley M (2001). Reviewing integrated science and mathematics: The search for evidence and definitions from new perspectives. School Science and Mathematics.

[CR23] Kelley TR, Knowles JG (2016). A conceptual framework for integrated STEM education. International Journal of STEM Education.

[CR24] LaForce M, Noble E, King H, Century J, Blackwell C, Holt S, Ibrahim A, Loo S (2016). The eight essential elements of inclusive STEM high schools. International Journal of STEM Education.

[CR25] Langdon D, McKittrick G, Beede D, Khan B, Doms M (2011). STEM: Good jobs now and for the future.

[CR26] Moore T, Smith K (2014). Advancing the state of the art of STEM integration. Journal of STEM Education.

[CR27] Moore T, Stohlmann M, Wang H, Tank K, Glancy A, Roehrig G, Purzer S, Strobel J, Cardella M (2014). Implementation and integration of engineering in K-12 STEM education. *Engineering in Pre-College Settings: Synthesizing Research, Policy, and Practices*.

[CR28] Nagle R, Gagnon S, Thomas A, Grimes J (2008). Best practices in planning and conducting needs assessments. *Best practices in school psychology V*.

[CR29] National Academies of Sciences, Engineering, and Medicine (2017). *Increasing the roles and significance of teachers in policymaking for K-12 engineering education: Proceedings of a convocation*.

[CR30] Peters MA (2006). Building knowledge cultures: Education and development in the age of knowledge capitalism.

[CR31] Roehrig GH, Wang H, Moore TJ, Park MS (2012). Is adding the “E” enough? Investigating the impact of K-12 engineering standards on the implementation of STEM integration. School Science and Mathematics.

[CR32] Romrell, D., Kidder, L. C., & Wood, E. (2014). The SAMR model as a framework for evaluating mLearning. *Online Learning, 18*(2). Accessed 20 April, 2017 from http://olj.onlinelearningconsortium.org/index.php/olj/article/view/435/105.

[CR33] Roth KJ, Garnier HE, Chen C, Lemmens M, Schwille K, Wickler NIZ (2011). Videobased lesson analysis: Effective science PD for teacher and student learning. Journal of Research in Science Teaching.

[CR34] Sanders M (2009). STEM, STEM education, STEMmania. The Technology Teacher.

[CR35] Sherin MG, Han SY (2004). Teacher learning in the context of a video club. Teaching and Teacher Education.

[CR36] Shernoff DJ (2013). Optimal learning environments to promote student engagement.

[CR37] Stohlmann M, Moore TJ, Roehrig GH (2012). Considerations for teaching integrated STEM education. Journal of Pre-College Engineering Education Research.

[CR38] The Royal Society Science Policy Centre (2014). Vision for science and mathematics education.

[CR39] Tillman D, An S, Cohen J, Kjellstrom W, Boren R (2014). Exploring wind power: Improving mathematical thinking through digital fabrication. Journal of Educational Multimedia and Hypermedia.

[CR40] US Department of Education, Office of Innovation and Improvement. (2016). *STEM 2026: A vision for innovation in STEM education*. Washington DC: Author

[CR41] Weis L, Eisenhart M, Cipollone K, Stich AE, Nikischer AB, Hanson J, Leibrandt SO, Allen CD, Dominguez R (2015). In the guise of STEM education reform: Opportunity structures and outcomes in inclusive STEM-focused high schools. American Educational Research Journal.

